# Kainic Acid-Induced Post-Status Epilepticus Models of Temporal Lobe Epilepsy with Diverging Seizure Phenotype and Neuropathology

**DOI:** 10.3389/fneur.2017.00588

**Published:** 2017-11-06

**Authors:** Daniele Bertoglio, Halima Amhaoul, Annemie Van Eetveldt, Ruben Houbrechts, Sebastiaan Van De Vijver, Idrish Ali, Stefanie Dedeurwaerdere

**Affiliations:** ^1^Department of Translational Neurosciences, University of Antwerp, Antwerp, Belgium

**Keywords:** epilepsy model, status epilepticus, spontaneous recurrent seizures, translocator protein, epileptogenesis, strain

## Abstract

The aim of epilepsy models is to investigate disease ontogenesis and therapeutic interventions in a consistent and prospective manner. The kainic acid-induced *status epilepticus* (KASE) rat model is a widely used, well-validated model for temporal lobe epilepsy (TLE). As we noted significant variability within the model between labs potentially related to the rat strain used, we aimed to describe two variants of this model with diverging seizure phenotype and neuropathology. In addition, we evaluated two different protocols to induce status epilepticus (SE). Wistar Han (Charles River, France) and Sprague-Dawley (Harlan, The Netherlands) rats were subjected to KASE using the Hellier kainic acid (KA) and a modified injection scheme. Duration of SE and latent phase were characterized by video-electroencephalography (vEEG) in a subgroup of animals, while animals were sacrificed 1 week (subacute phase) and 12 weeks (chronic phase) post-SE. In the 12 weeks post-SE groups, seizures were monitored with vEEG. Neuronal loss (neuronal nuclei), microglial activation (OX-42 and translocator protein), and neurodegeneration (Fluorojade C) were assessed. First, the Hellier protocol caused very high mortality in WH/CR rats compared to SD/H animals. The modified protocol resulted in a similar SE severity for WH/CR and SD/H rats, but effectively improved survival rates. The latent phase was significantly shorter (*p* < 0.0001) in SD/H (median 8.3 days) animals compared to WH/CR (median 15.4 days). During the chronic phase, SD/H rats had more seizures/day compared to WH/CR animals (*p* < 0.01). However, neuronal degeneration and cell loss were overall more extensive in WH/CR than in SD/H rats; microglia activation was similar between the two strains 1 week post-SE, but higher in WH/CR rats 12 weeks post-SE. These neuropathological differences may be more related to the distinct neurotoxic effects of KA in the two rat strains than being the outcome of seizure burden itself. The divergences in disease progression and seizure outcome, in addition to the histopathological dissimilarities, further substantiate the existence of strain differences for the KASE rat model of TLE.

## Introduction

Animal models are very important for biomedical researchers to investigate disease ontogenesis or to evaluate pharmacological interventions. They have been essential tools in the discovery of antiepileptic drugs (AEDs) (1, 2). Moreover, chronic epilepsy models with high etiological relevance, such as the *status epilepticus* (SE), febrile seizure, and traumatic brain injury models, have played an important role in disentangling the pathophysiological processes involved in human epilepsy (1, 3–5). The advantage of these chronic models is that they enable to study epileptogenesis in a consistent and prospective manner, which is almost impossible to accomplish in patients due to heterogeneity of disease ontogenesis and interfering factors, such as antiepileptic medication, during clinical studies. In addition, models provide the possibility to test new drug targets for disease-modifying potential, a needed paradigm shift in respect to the current availability of symptomatic AEDs only.

Careful consideration in the selection of one or the other animal model is required. This choice depends on several factors, including the epilepsy subtype to be modeled as well as the research aims. The kainic acid-induced *status epilepticus* (KASE) model is a well-validated model of temporal lobe epilepsy (TLE) (6). TLE is the most common form of focal epilepsy in adults and often refractory epilepsy to medication (7). The KASE model involves induction of SE, which is characterized by a continuous seizure activity or as a series of seizures without retaining full consciousness in between (8). In the majority of animals, this subsequently initiates a process of epileptogenesis, which later leads to the development of spontaneous epileptic seizures. The model allows studying the different stages between SE and the development of TLE including SE, the acute and latent stages of epileptogenesis and chronic epilepsy. TLE models reflect similar neuropathological characteristics as seen in patients with TLE including mild (MRI negative) to severe structural changes such as hippocampal sclerosis (HS) (MRI positive) (9–11), accompanied by several processes such as cell loss and inflammation. HS is a common pathology in patients with mesial TLE (approximately 65% of patients) and can be classified into typical (type 1) and atypical (type 2 and 3), based on the histological patterns of neuronal loss and gliosis (12). Nearly 60–80% of patients with mesial TLE are affected by type 1 HS, which is characterized by neuronal loss predominantly in CA1 but also CA4 and CA3. Accordingly, in the KASE rat model of TLE, a high proportion of animals is characterized by a histological pattern similar to type 1 HS in patients (13).

At our laboratory, the KASE model is used to study the role of brain inflammation as well as its association with chronic seizure burden in epileptic rats (14). Earlier experiments in our group utilized Wistar Han rats from Charles River (WH/CR; France) (14, 15). We noticed that across different laboratories, Wistar Han rats have a relatively low number (on average <1/day) of spontaneous recurrent seizures (SRS) during the chronic period (11, 13–16), and extensive microgliosis and neurodegeneration (13, 15, 17, 18). Nevertheless, studies in models with low seizure frequency or an overall lower susceptibility to develop seizures after an epileptogenic insult are very useful in investigating molecular, epigenetic, and neurobiological alterations associated with epileptogenesis and seizure susceptibility. Such models could provide important insights in the identification and evaluation of risk and precipitating factors, which could otherwise be less evident. Besides, these models could be useful in the evaluation of anti-epileptogenic treatments interfering with epileptogenic mechanisms to delay or prevent development of epilepsy.

In addition to striving for a better understanding of epileptogenesis, an important part of epilepsy research is to find new treatments. For evaluating novel antiepileptic treatments, a model with higher seizure burden could be attractive. This would allow detecting more subtle effects of the treatment on the number and severity of SRS (instead of an all-or-none effect). An extensive literature search indicated that Sprague-Dawley rats from the Harlan Laboratories (SD/H; The Netherlands) have frequent SRS during the chronic period when subjected to KASE (17, 19–22).

With this study, we aimed to describe two rat strains subjected to KASE that express either a low or a high prevalence of SRS during the chronic period using the same initial trigger, i.e., KASE. A second aim was to investigate neuropathological differences (neurodegeneration and cell loss, and microgliosis) during both subacute and chronic stages of KASE-induced TLE. To fulfill these goals, we first compared two protocols for the induction of SE as described by and modified from Hellier (8) [5 mg/kg every hour and 2.5 mg/kg every 30 min kainic acid (KA), respectively] to assess the vulnerability of the two strains to KA. Second, seizure phenotype was determined by means of continuous (24 h/day) video-electroencephalography (vEEG) and neuropathological changes with *postmortem* analysis, respectively.

## Materials and Methods

### Animal Ethics and Care

Male WH and SD rats were bought from Charles River (France) and Harlan Laboratories (The Netherlands), respectively. WH/CR were the standardly used animals at the laboratory, while SD/H were chosen due to their high chronic seizure frequency reported in the literature. All rats were single housed under a 12 h light/dark cycle, in a temperature (22 ± 2°C) and humidity (55 ± 10%) controlled environment. Food and water were available *ad libitum*. Animals were allowed 6 days of acclimatization to the animal facilities before the start of the experiments and were treated in accordance with the guidelines approved by the European Directive (2010/63/EU) on the protection of animals used for experimental and other scientific purposes. All animal experiments were approved (ECD 2014-39) by the ethical committee of the University of Antwerp (Belgium).

### Study Design

**Figure 1:**
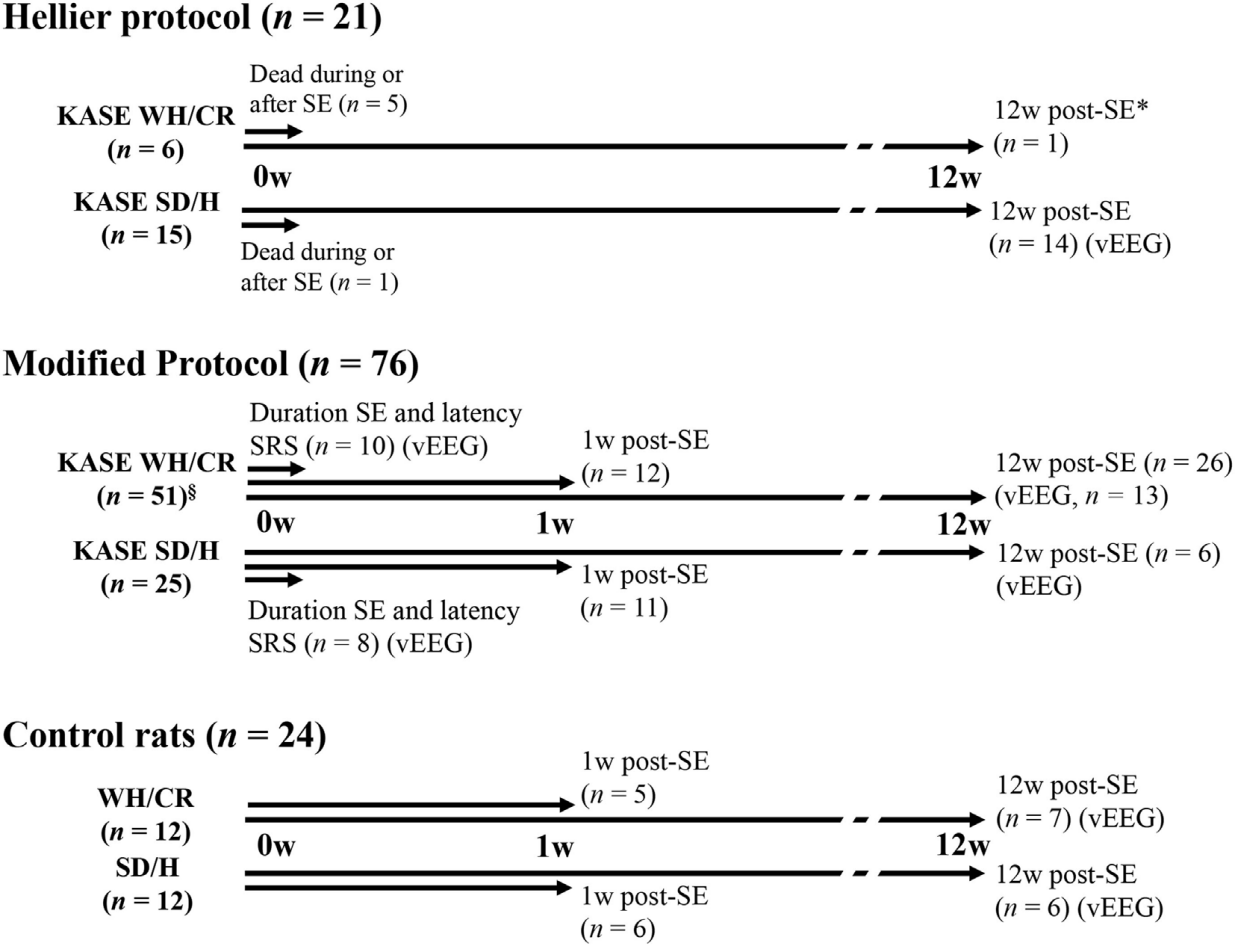
Schematic diagram illustrating the experimental design, the number of animals used for each SE induction protocol, and strain. Three animal groups were included in the study: a group of animals (duration SE and latency SRS) was vEEG monitored to determine the duration of SE and the latency to the first SRS (modified protocol: KASE WH/CR, *n* = 10; KASE SD/H, *n* = 8). A second group of animals (1 w post-SE) was sacrificed at subacute phase for *postmortem* analysis (modified protocol: KASE WH/CR, *n* = 12; KASE SD/H, *n* = 11; control rats: WH/CR, *n* = 5; SD/H, *n* = 6). Finally, a third group of animals (12 w post-SE) was vEEG monitored during chronic epilepsy and sacrificed for *postmortem* analysis (Hellier protocol: KASE SD/H, *n* = 14, only vEEG; modified protocol: KASE WH/CR, *n* = 26; SD/H, *n* = 6; control rats: WH/CR, *n* = 7; SD/H, *n* = 6). vEEG indicates animal that underwent vEEG monitoring. * was not included for further vEEG analysis or *postmortem* evaluation. ^§^Three WH/CR died during SE. vEEG indicates animals underwent electrode implantation and were vEEG monitored. SE, *status epilepticus*; w, weeks; KASE, kainic acid-induced *status epilepticus*; WH/CR, Wistar Han/Charles River; SD/H, Sprague-Dawley/Harlan; *n*, number; vEEG, video-electroencephalography; SRS, spontaneous recurrent seizures.

A total of 121 animals were included in the study from which 97 rats (WH/CR, *n* = 57; SD/H, *n* = 40) were subjected to the multiple, low-dose injection KASE model. The other 24 animals represented the control group. In a first step, the severity and mortality of SE were compared between the Hellier protocol (8) and a modified protocol as we noticed that the WH/CR were strikingly sensitive to the standard Hellier protocol. As there was a high mortality in the WH/CR strain after SE induced by the Hellier protocol, vEEG and histopathological evaluations were limited to animals subjected to the modified protocol. In addition, a control group (*n* = 5–7) for each strain and each time point was also included. The study design is summarized in Figure 1: a group of animals was used to evaluate SE duration and the latency to the first SRS by means of vEEG recording, a second group was sacrificed 1 week post-SE (subacute phase), while a third group of animals was followed up longitudinally until the chronic period (12 weeks post-SE) to determine SRS outcome by means of vEEG recording. Differences in the two strains were evaluated at each of the time points investigated.

### Induction of SE

At SE induction, the rats were 7.5 weeks old with an average weight of 231.3 ± 1.8 g for WH/CR and 238.4 ± 2.3 g for SD/H rats. In a first group of animals (WH/CR *n* = 6; SD/H *n* = 15), the Hellier protocol was used to induce SE (8). Briefly, they received an initial dose of 5 mg/kg KA [subcutaneously (s.c.); sourced from A.G. Scientific, USA for all experiments], repeated every hour until the animals displayed four convulsive seizures (type S4–S5) (Table S1 in Supplementary Material). To assure SE was induced, the animal had to experience four convulsive seizures within 1 h. This protocol is referred to as the Hellier protocol (8). The modified protocol was based on previous studies in which repeated doses of KA have been tapered down to 2.5 mg/kg in Wistar Han rats to improve survival and welfare requirements from local ethical committees (11, 23, 24). The protocol presented here results from optimizing its efficiency regarding epilepsy outcomes and to provide excellent survival rate at the same time. The modified protocol (WH/CR *n* = 51; SD/H *n* = 25) involved an initial dose of 7.5 mg/kg. After 45 min, repetitive injections of 2.5 mg/kg were given every 30 min (14). Injections were repeated until convulsive seizures were induced and then stopped to avoid overdose. According to the protocol, if an animal did not present four convulsive seizures within 1 h, injections would be continued. However, it was never required since all animals reached the criterion. After 4 h of SE (induction score ≥3), diazepam (4 mg/kg, i.p.; NV Roche SA, Belgium) was given to reduce the mortality, although this dose did not terminate SE (24). For both protocols, the first convulsive seizure was considered as the start of SE. The number of injections was limited to a maximum of 10, although rarely more than 8 injections were needed, and all animals entered SE. Hartmann’s solution (10 ml/kg, s.c.) was administered to prevent dehydration (15). All control animals (*n* = 24) received saline injections only (range of 3–6 injections, s.c.). Additional care was taken the days following SE by providing the animals with enriched soft food pellets, manual feeding, and Hartmann’s solution (10 ml/kg, s.c.) if required (15).

Several induction variables were determined for statistical analysis including the induction score, which represented the severity of the SE (score ranging from 0 to 7, with 0 indicating no SE and 7 a very severe SE) (Table S2 in Supplementary Material), sickness behavior, and body weight change (%). Sickness behavior was determined as composite score of different criteria, where for each criterion (namely feeding and drinking, mobility, visible stress or discomfort, signs of pain, respiration, fur, and body weight) a score from 0 (normal) to 2 (highly abnormal) was given. Intermediate scores were included when only a mild change was visible. The sum of all criteria defined the composite score.

### Evaluation of SE and SRS by Means of vEEG

To determine the duration of SE and the latency to first SRS, animals (WH/CR *n* = 10; SD/H *n* = 8) were implanted with epidural electrodes 2 weeks before the induction of SE with modified protocol as previously described (14). After 4 h of observation of SE, the animals were connected to the vEEG system (14). From the EEG, we considered the SE terminated when the last high-amplitude periodic epileptiform discharge occurred as previously described (14, 21).

To investigate SRS during chronic epilepsy, KASE animals (modified protocol: WH/CR *n* = 13; SD/H *n* = 6; Hellier protocol: SD/H *n* = 14) were followed up with vEEG for a period of 14 days. The implantation of the epidural electrodes was performed 8 weeks after SE to provide the animals with a recovery period of at least 2 weeks before the start of the recording. The first day of vEEG recording was excluded from the analysis to allow the animals to acclimatize to the vEEG setup. Analysis of vEEG recordings was performed manually using Neuroscore 3.0 (Data Sciences International, USA) by an experienced investigator. The identification of SRS was executed as previously described (25). The severity of SRS was determined according to the modified scale of Racine (26). Duration of SE, latency to the first SRS, duration of SRS, severity of SRS, circadian distribution of SRS, and number of SRS were determined for each strain following SE induction with the modified protocol.

### Tissue Collection

All animals were sacrificed by rapid decapitation. Brains were immediately removed from the skull and directly fresh-frozen in 2-methylbutane at −35°C and further preserved at −80°C. Serial coronal sections (20 µm of thickness) were collected for analysis starting at −3.0 mm from bregma (dorsal hippocampus; Figure S1 in Supplementary Material) (27) until −3.6 mm from bregma, in triplicate, on positively charged glass slides (Menzel-Gläser, Thermo Fischer Scientific, USA) using a cryostat (Leica, Germany).

### Histopathology

Neuronal nuclei (NeuN), OX-42, and Fluorojade C (FjC) staining were performed as previously described (15, 25, 28) to visualize neurons, microglial activation, and degenerating neurons, respectively. Staining was quantified in the hippocampal sub-regions cornu ammonis 1, 3, 4, and dentate hilus (DH), and in the piriform cortex (PC) (Figure S1 in Supplementary Material). These regions were chosen for histopathological analysis given their implication in TLE. They are highly interconnected with other limbic nuclei, providing the substrate for the hypersynchrony and hyperexcitability associated with seizure generation and propagation.

Neuronal nuclei and OX-42 immunostaining were performed for neuronal loss and microglia activation, respectively. Briefly, after fixating and blocking, sections were incubated overnight with the primary antibody [mouse anti-rat NeuN, 1:2.000, Merck Millipore, Germany or mouse anti-rat OX-42 (CD11b), 1:1.000, AbD Serotec, UK] in blocking solution. The next morning sections were incubated with peroxidase-conjugated secondary antibody (donkey anti-mouse IgG-HRP, 1:500, Jackson Immunoresearch, UK) in antibody diluent. Finally, to visualize the binding, sections were exposed for 10 min to the colorimetric diaminobenzidine staining.

Fluorojade C staining was performed to visualize degenerating neurons. Briefly, sections were first immersed in a solution consisting of 1% sodium hydroxide in 80% ethanol for 5 min, then rinsed for 2 min in 70% ethanol, 2 min in distilled water, and incubated in 0.06% potassium permanganate solution for 10 min. After 5 min in distilled water, sections were transferred to a 0.0005% solution of FjC (Merck Millipore, Germany) dissolved in 0.1% acetic acid vehicle. Following three 2 min rinses in distilled water, the sections were dehydrated and cleared in xylene for at least 1 min and then coverslipped.

Quantification of the number of neurons was performed using ImageJ software (National Institute of Health, USA) as previous described (29) in CA1, CA3, CA4, DH, and PC (Figure S1 in Supplementary Material). The intensity threshold and the minimum and maximum cell size parameter values were initially determined in an empirical manner under blind conditions. Automatic quantification was done blinded for treatment on triplicate sections of which the mean score was used for statistical analysis.

Area of increased OX-42 staining was quantitatively evaluated in the same regions as for NeuN staining using ImageJ software as previously described (13). FjC-positive cells were manually counted by two investigators blinded to treatment in the same regions used for the other variables (Figure S1 in Supplementary Material). Quantification was performed on triplicate sections of which the mean score was used for statistical analysis.

### Autoradiography

Translocator protein (TSPO) expression was determined by *in vitro* autoradiography with ^3^H-PK11195 (PerkinElmer, USA) as previously described (15). This is a valuable tool as it has potential translational clinical use due to the availability of TSPO PET tracers for non-invasive imaging. The TSPO focal binding (TSPO binding in the focal region; Bq/mg tissue) and the TSPO% area (area with increased TSPO) were determined as previously described (15) for the KASE animals. TSPO expression was quantitatively measured in the following ROIs: CA1, CA3, CA4, DH, and PC (Figure S1 in Supplementary Material). The specific binding of the radioligand (TSPO-specific binding; Bq/mg tissue) was determined for the control and KASE groups. In addition, the TSPO focal binding (TSPO binding in the focal region; Bq/mg tissue) and the TSPO% area (area with increased TSPO) were determined as previously described for the KASE animals (15, 25).

### Statistical Analysis

The Kolmogorov–Smirnov test showed that not all data were normally distributed and, therefore, it was decided to use non-parametric tests for the analyses. A Mann–Whitney *U* test was used to compare the two KASE groups (KASE WH/CR and KASE SD/H), whereas a Kruskal–Wallis test with *post hoc* Dunn’s multiple comparisons test to evaluate the four different groups at once (namely, the control WH/CR, KASE WH/CR, control SD/H, and KASE SD/H groups). The Fischer’s exact test was used to compare severity of SRS and circadian distribution of SRS between KASE groups. Spearman’s rank test was used to evaluate correlations. Data from the two time points were analyzed separately. Data are represented as box plot and reported as median ± interquartile ranges (IQRs), unless specified. GraphPad Prism 6 (GraphPad Software, USA) was used for all analyses. Tests were two-tailed and statistical significance was set at *p* < 0.05.

## Results

### Mortality, Severity, and Duration of the KA-Induced SE

**Figure 2:**
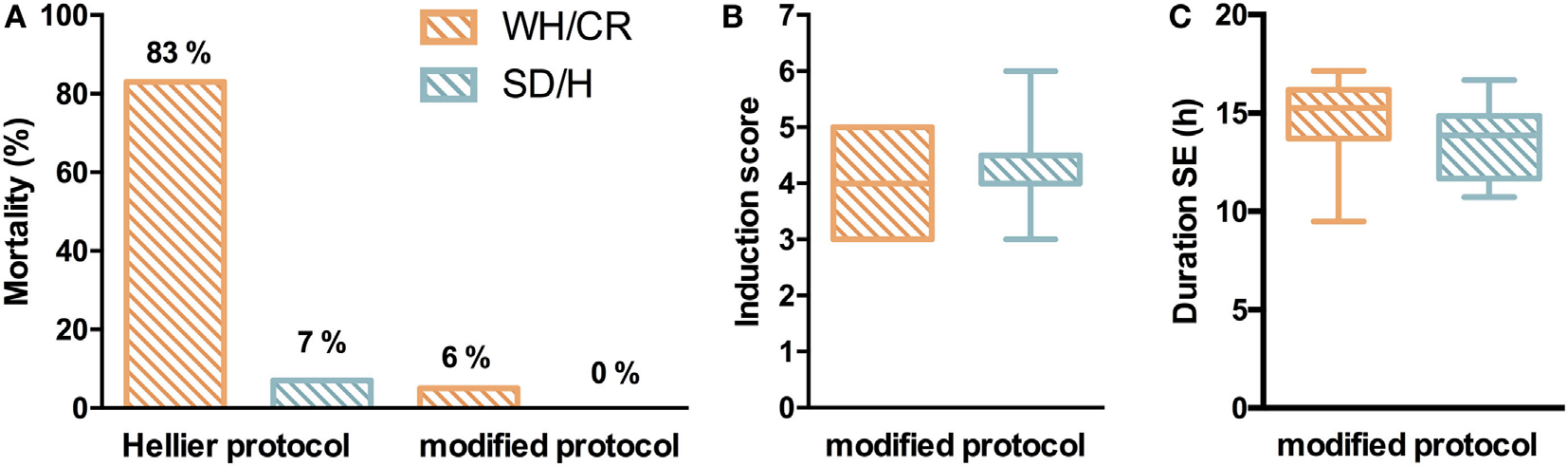
Status epilepticus (SE) in KASE WH/CR and KASE SD/H rats. **(A)** Mortality rates for both rat strains during SE induction. **(B)** Severity of the SE between the two strains during the modified protocol. Hellier protocol: WH/CR *n* = 6 and SD/H *n* = 15; modified protocol: WH/CR *n* = 51 and SD/H *n* = 25 **(A,B)**. **(C)** Duration of SE in WH/CR (*n* = 10) and SD/H (*n* = 8) during the modified protocol. WH/CR, Wistar Han/Charles River; SD/H, Sprague-Dawley/Harlan; KASE, kainic acid-induced *status epilepticus*.

Two similar SE induction protocols were evaluated in both the WH/CR and SD/H strains to compare their responsiveness to each protocol. Following Hellier protocol, all SD/H rats went into SE for at least 4 h without any mortality, and only 1/15 animal did not recover from the SE and died 2 days post-SE (7% mortality) (Figure 2A). Instead, four WH/CR rats died within 4 h from the induction of SE and one rat died due to SE-related sickness 3 days post-SE (83% mortality). The considerable mortality associated with the Hellier protocol in the WH/CR rats underlines the need for a different SE induction protocol in this strain. While using the modified protocol, none of the SD/H rats (*n* = 25) died during the induction of SE (0% mortality) and only 3/51 WH/CR rats died during or the days following induction of SE (6% mortality) (Figure 2A). No significant difference was found in the induction score of the SE between WH/CR and SD/H when using the modified protocol (Figure 2B). The duration of SE was evaluated by means of vEEG monitoring in a subset of animals and no difference between strains could be found [WH/CR = 15.26 h (IQR = 13.72–16.19 h); SD/H = 13.87 h (IQR = 11.68–14.86 h)] (Figure 2C). During post-SE follow-up, strains did not display any significant difference in sickness behavior [sickness score: WH/CR = 4 (IQR = 3–6.5), while SD/H = 4.5 (IQR = 3–7.5)] or body weight change 1 day post-SE (WH/CR = −14.9 ± 3.2%, while SD/H = −14.3 ± 3.6%, compared to the day of induction of SE). All animals fully recovered the weight loss within 4 days’ post-SE. No differences were seen in SD/H animals between the two protocols for any of the parameters evaluated.

### The Two Strains Are Characterized by Different SRS Outcome

**Figure 3:**
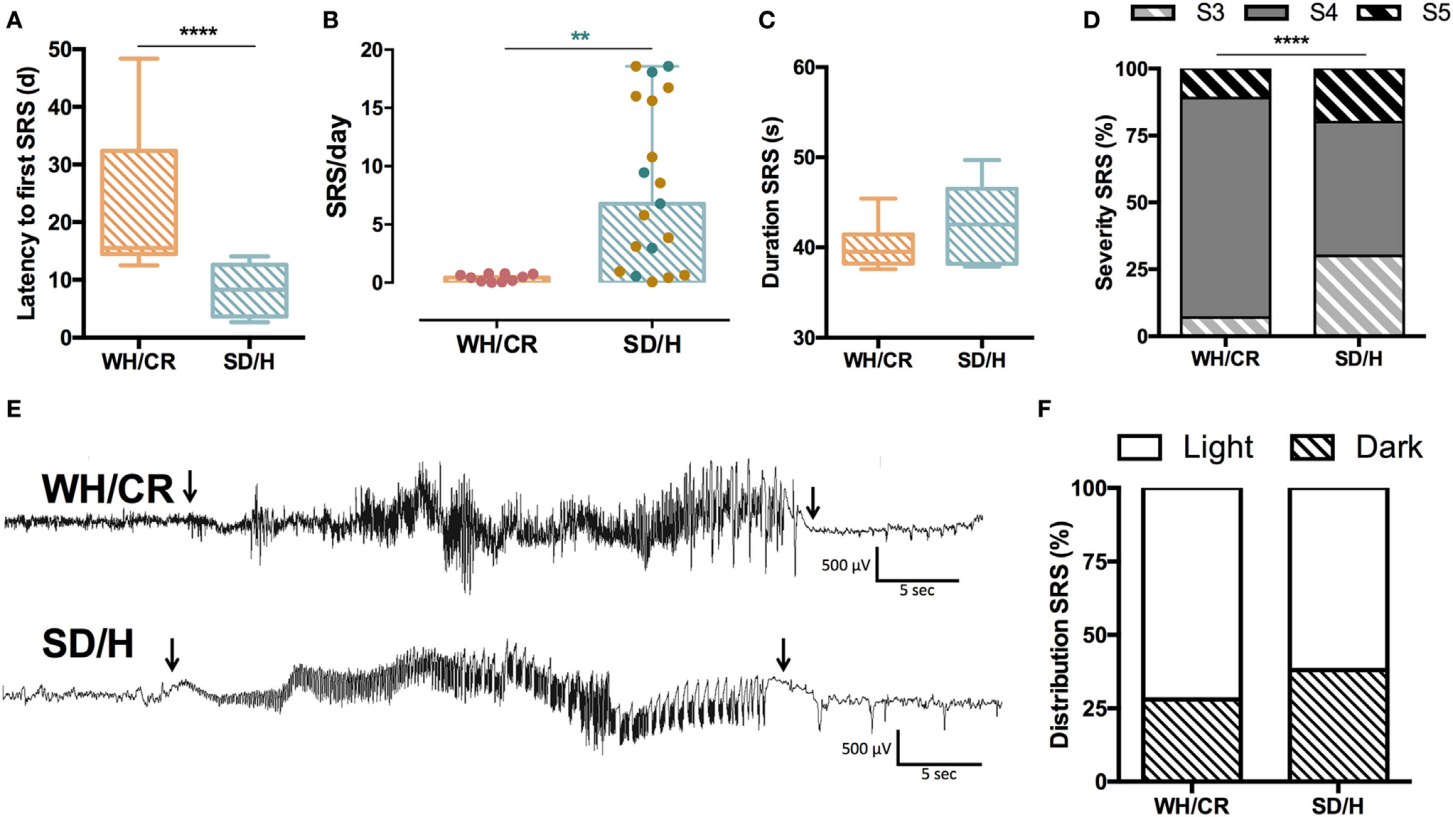
SRS characterization in kainic acid-induced SE (KASE) WH/CR and KASE SD/H rats. **(A)** WH/CR rats (*n* = 10) showed a significant longer latency to the first SRS when compared to SD/H (*n* = 8). Mann–Whitney *U* test. ****p < 0.0001. **(B)** SRS frequency during the chronic period. A significant higher number of SRS per day was demonstrated for the SD/H rats compared to the WH/CR rats 12 weeks post-status epilepticus (SE). Red and blue dots represent WH/CR and SD/H, respectively, following modified protocol; brown dots represent SD/H following Hellier protocol. Mann–Whitney *U* test. **p < 0.01 after considering only animals that underwent modified SE protocol. **(C)** The duration of SRS was similar in both strains. **(D)** WH/CR and SD/H were characterized by different distribution of SRS. ****p < 0.0001. Fischer’s exact test. **(E)** Representative spontaneous electrographic seizure (S4) during chronic period (12 weeks post-SE) of a WH/CR and SD/H rat. Arrows indicate the onset and the end of the seizure. **(F)** The circadian distribution of SRS was similar in both strains with a higher frequency of SRS during the light phase. WH/CR *n* = 10 and SD/H *n* = 6 **(C,D,F)**. WH/CR, Wistar Han/Charles River; SD/H, Sprague-Dawley/Harlan; SRS, spontaneous recurrent seizures; d, days. Graph B is reported as mean ± SD.

For a cohort of animals, vEEG monitoring was started at SE to determine the duration of SE and the latency to the first SRS. These animals underwent surgical implantation of the recording electrodes 2 weeks before SE. WH/CR rats experienced a significantly longer latency to the first SRS compared to SD/H animals [WH/CR = 15.4 days (IQR = 14.5–32.4), SD/H = 8.3 days (IQR = 3.7–12.6); *p* < 0.0001] (Figure 3A). In the cohort of animals allocated for the vEEG monitoring during chronic epilepsy, the surgical implantation of the recording electrodes occurred 6 weeks post-SE. This was followed by a 2 weeks recovery before the start of the recording. During vEEG monitoring in the chronic phase, 10 out of 13 KASE WH/CR rats experienced SRS, while all KASE SD/H rats did following both protocols (Hellier protocol = 14/14, modified protocol = 6/6). To compare SRS outcome parameters side-by-side between the two strains, only data following the modified protocol were included. After excluding the non-seizing KASE WH/CR rats, the number of SRS per day was still significantly lower compared to KASE SD/H rats [WH/CR = 0.46 SRS/day (IQR = 0.12–0.75), SD/H = 6.78 SRS/day (IQR = 0.96–17.98); *p* < 0.01] (Figures 3B,E). The average duration of SRS was similar in the two strains [WH/CR = 39.5 s (IQR = 38.2–41.4 s), SD/H = 42.5 s (IQR = 38.2–46.5 s)] (Figure 3C), whereas the SRS severity was significantly different between the two strains (*p* < 0.0001) (Figure 3D). Finally, both strains displayed a similar SRS distribution between light phase (WH/CR = 72%; SD/H = 62%) and dark phase (WH/CR = 28%; SD/H = 38%) of the day (Figure 3F).

### Neuronal Loss Is More Extensive in KASE WH/CR Rats than KASE SD/H Rats

**Figure 4:**
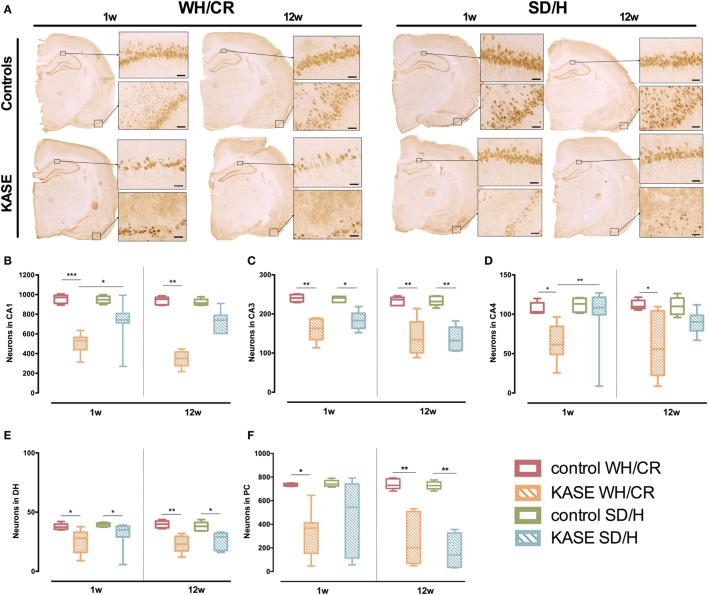
Neuronal loss in KASE WH/CR and KASE SD/H rats. **(A)** Representative KASE and control rats 1 and 12 weeks post-status epilepticus (SE). At both time points, a significant difference could be demonstrated for the investigated regions **(B–F)** between the respective control and KASE WH/CR animals, while for the SD/H animals only in CA3 and DH 1 week post-SE, and CA3, DH, and PC 12 weeks post-SE **(C,E,F)**. In addition, for the CA1 and CA4 sub-regions, significant higher neuronal loss could be demonstrated for the KASE WH/CR animals compared to KASE SD/H rats 1 week post-SE. Kruskal–Wallis test with *post hoc* Dunn’s test **(B–F)**. 1 week post-SE: control WH/CR *n* = 5, KASE WH/CR *n* = 10, control SD/H *n* = 5 and KASE SD/H *n* = 9; 12 weeks post-SE: control WH/CR *n* = 7, KASE WH/CR *n* = 6, control SD/H *n* = 6 and KASE SD/H *n* = 6. Insets were taken at 10× magnification. Scale bar = 50 µm. *p < 0.05, **p < 0.01. WH/CR, Wistar Han/Charles River; SD/H, Sprague-Dawley/Harlan; w, week; CA, cornu ammonis; DH, dentate hilus; PC, piriform cortex; KASE, kainic acid-induced *status epilepticus*.

One week post-SE significant neuronal loss was present in all hippocampal sub-regions and PC in KASE WH/CR rats in comparison to control rats (*p* < 0.05) (Figure 4). On the contrary, in the SD/H rats, significant neuronal loss was found only in CA3 and DH. In addition, KASE WH/CR rats showed significant neuronal loss in CA1 and CA4 compared to KASE SD/H animals (*p* < 0.05).

Twelve weeks post-SE, significant neuronal loss was observed for all investigated regions in KASE WH/CR compared to the control rats (*p* < 0.05) (Figure 4), while significant neuronal loss was limited to CA3, DH, and PC in KASE SD/H rats compared to the controls (*p* < 0.05).

### KASE WH/CR and SD/H Rats Show Similar OX-42 Immunoreactivity Only at Subacute Phase

**Figure 5:**
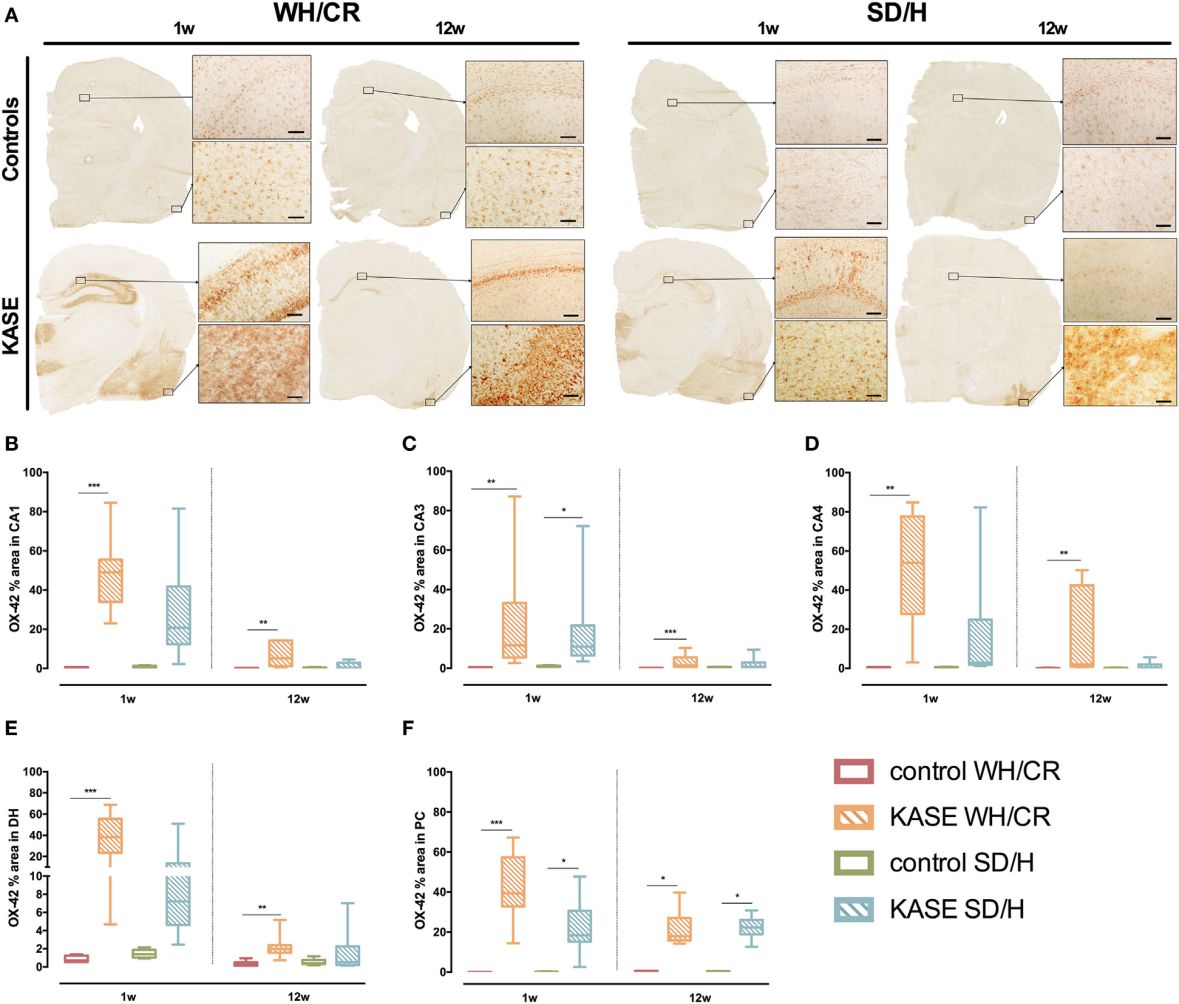
Microglial activation in KASE WH/CR and KASE SD/H rats. **(A)** Representative KASE and control rats 1 and 12 weeks post-status epilepticus (SE). For all investigated regions, a significant difference could be demonstrated between WH/CR controls and KASE rats 1 week post-SE as well as 12 weeks post-SE **(B–F)**. For the SD/H rats, this was only observed for the CA3 sub-region of the hippocampus and PC 1 week post-SE, while only in PC during the chronic period. Kruskal–Wallis test with *post hoc* Dunn’s test **(B–F)**. *p < 0.05, **p < 0.01, ***p < 0.001. 1 week post-SE: control WH/CR *n* = 4–5, KASE WH/CR *n* = 8–10, control SD/H *n* = 5 and KASE SD/H *n* = 6; 12 weeks post-SE: control WH/CR *n* = 5–6, KASE WH/CR *n* = 7, control SD/H *n* = 4–6 and KASE SD/H *n* = 6–8. Insets were taken at 10× magnification. Scale bar = 100 µm. WH/CR, Wistar Han/Charles River; SD/H, Sprague-Dawley/Harlan; w, week; CA, cornu ammonis; DH, dentate hilus; PC, piriform cortex; KASE, kainic acid-induced *status epilepticus*.

Kainic acid-induced *status epilepticus* WH/CR rats displayed increased OX-42 immunoreactivity compared to controls in hippocampal sub-regions and PC 1 week post-SE (*p* < 0.01) (Figure 5). In KASE SD/H rats, only CA3 and PC were significantly increased compared to control SD/H rats (*p* < 0.05) (Figure 5).

Twelve weeks post-SE microglial activation was relatively less evident, however, it was still significantly increased in KASE WH/CR animals compared to controls in all the investigated regions (*p* < 0.01) (Figure 5). On the other hand, in KASE SD/H animals, increased OX-42 immunoreactivity was confined to PC (*p* < 0.05) (Figure 5F).

### TSPO Displays Different Upregulation Patterns in KASE WH/CR and SD/H Rats

**Figure 6:**
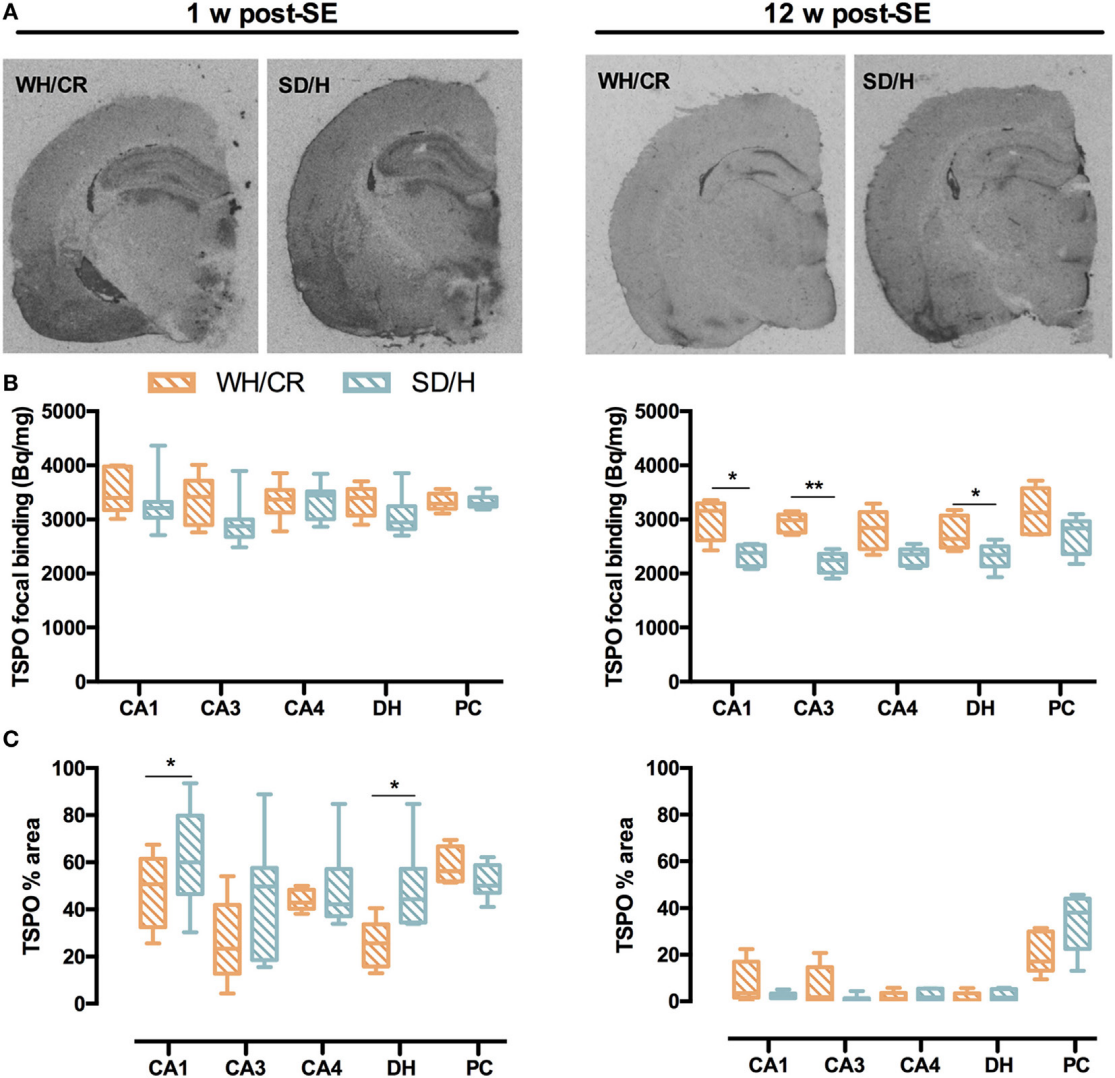
TSPO focal binding and % area in WH/CR and SD/H rats. **(A)** Representative autoradiograms showing TSPO expression 1 and 12 weeks post-status epilepticus (SE). **(B)** TSPO focal binding in the subacute phase was similar for the two strains, while in the chronic phase, it was higher for the KASE WH/CR rats for all the brain regions except for the CA4 and PC when compared to KASE SD/H animals. **(C)** The TSPO% area was significantly increased in the KASE SD/H animals in the CA1 and DH when compared to the KASE WH/CR rats 1 week post-SE. Twelve weeks post-SE no differences could be determined. Mann–Whitney *U* test **(B,C)**. *p < 0.05, **p < 0.01. KASE WH/CR *n* = 5–7 and KASE SD/H *n* = 6. TSPO, translocator protein; WH/CR, Wistar Han/Charles River; SD/H, Sprague-Dawley/Harlan; w, week; CA, cornu ammonis; DH, dentate hilus; PC, piriform cortex; KASE, kainic acid-induced *status epilepticus*.

During subacute phase, KASE animals of both strains had significantly increased TSPO-specific binding in all evaluated regions compared to controls (*p* < 0.05) (Figure S2 in Supplementary Material). Accordingly, the TSPO focal binding was similar between the two KASE groups (Figures 6A,B), whereas TSPO% area was significantly increased in KASE SD/H rats compared to KASE WH/CR in CA1 and DH (*p* < 0.05) (Figure 6C).

Translocator protein upregulation was less evident 12 weeks post-SE, nevertheless all evaluated brain regions were increased in KASE SD/H compared to control SD/H rats (*p* < 0.01) and a significant increase in TSPO-specific binding was also demonstrated in the CA1 of KASE WH/CR rats compared to controls (*p* < 0.05) (Figure S3 in Supplementary Material). Interestingly, TSPO focal binding was significantly higher in KASE WH/CR rats compared to KASE SD/H animals in CA1, CA3, and DH (*p* < 0.05) (Figure 6B), whereas TSPO% area between the two KASE groups did not differ in any investigated region (Figure 6C).

### Neurodegeneration Is More Extensive in KASE WH/CR than KASE SD/H Rats and Continues in the Chronic Phase Reflecting Neuronal Loss and Microglial Activation

**Figure 7:**
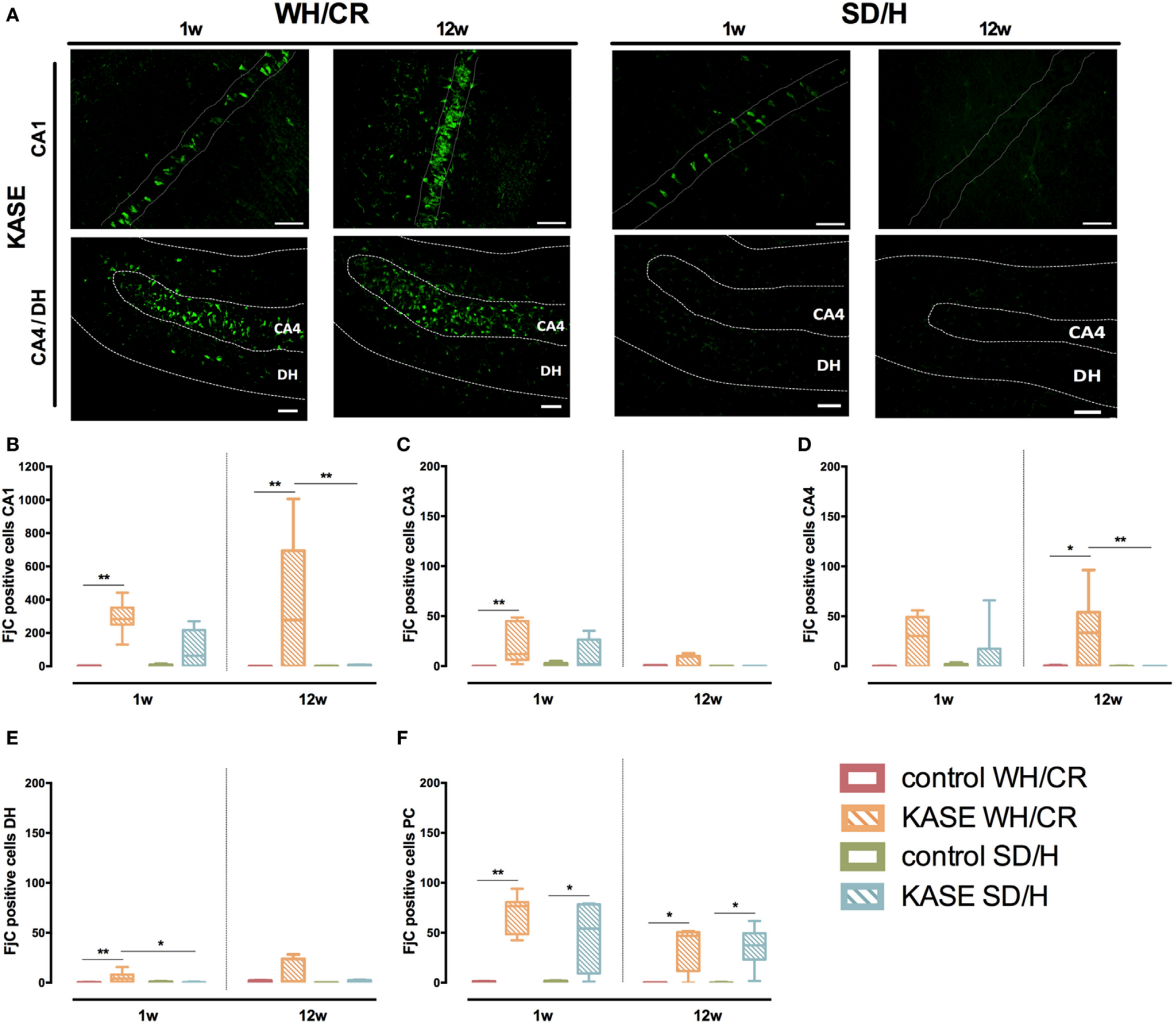
Neurodegeneration in KASE WH/CR and KASE SD/H rats. **(A)** Representative CA1 and CA4/DH images for KASE rats 1 and 12 weeks post-status epilepticus (SE). During both subacute and chronic phases, KASE WH/CR displayed an overall more extensive neurodegeneration than KASE SD/H in the investigated regions **(B–F)**. Kruskal–Wallis test with *post hoc* Dunn’s test **(B–F)**. *p < 0.05, **p < 0.01. One week post-SE: control WH/CR *n* = 4–5, KASE WH/CR *n* = 8–10, control SD/H *n* = 5 and KASE SD/H *n* = 6; 12 weeks post-SE: control WH/CR *n* = 5–6, KASE WH/CR *n* = 7, control SD/H *n* = 4–6 and KASE SD/H *n* = 6. Representative images: CA1 = 40× magnification, CA4/DH = 20× magnification. Scale bar = 50 µm. WH/CR, Wistar Han/Charles River; SD/H, Sprague-Dawley/Harlan; w, week; CA, cornu ammonis; DH, dentate hilus; PC, piriform cortex; KASE, kainic acid-induced *status epilepticus*.

During the subacute phase, KASE WH/CR rats displayed significant neurodegeneration in CA1, CA3, DH, and PC compared to controls (*p* < 0.01), while for the KASE SD/H animals, this was observed only in PC (*p* < 0.05) (Figure 7). In addition, KASE WH/CR displayed a significantly higher number of FjC positive neurons in DH compared to KASE SD/H (*p* < 0.05). The number of FjC-positive cells during the subacute phase inversely correlated with the number of neurons in all investigated regions (*R* = −0.46 to −0.77; *p* < 0.05) (Figure S4 in Supplementary Material). In addition, the number of FjC-positive cells during the subacute phase correlated with the % area of OX-42 immunoreactivity in all investigated regions (*R* = 0.55 to 0.94; *p* < 0.01) (Figure S5 in Supplementary Material).

Kainic acid-induced *status epilepticus* WH/CR animals displayed ongoing neurodegeneration even during the chronic epilepsy period in the CA1, CA4, and PC when compared to the controls (*p* < 0.05). Whereas for the SD/H animals, neurodegeneration was limited to the PC at the chronic phase (*p* < 0.05) (Figure 7). Accordingly, in CA1 and CA4 sub-regions, a significant higher number of degenerative neurons was observed for the KASE WH/CR animals compared to KASE SD/H rats (*p* < 0.05).

## Discussion

In this study, we describe two rat strains subjected to the KASE model and their differences in seizure phenotype and neuropathology. We also compared the Hellier protocol and a modified milder protocol for SE induction to determine the most suitable protocol in terms of mortality.

Using the Hellier protocol to induce SE led to high mortality in the WH/CR rats, which underlined the impossibility to use such protocol in this rat strain. When continuing with the modified protocol (2.5 mg/kg for repeated injections), this was well tolerated by both WH/CR and SD/H rats and resulted in a SE with similar severity and duration. This modified protocol allowed to substantially increase the survival of WH/CR rats without affecting the SE, thus reducing the overall number of animals needed in agreement with the refinement and reduction principles of the *in vivo* research.

Although only timing and dose of the repeated KA injections differed between the Hellier and our modified protocol, these results seem to suggest that the WH/CR rats handle KA differently than SD/H rats, which showed no/minimal mortality with both protocols. The large difference in mortality rate between the WH/CR and SD/H rats may be due to dissimilarities in pharmacokinetics and pharmacodynamics of KA in the WH/CR and SD/H rats. Indeed, WH rats are reported to be more sensitive to the excitotoxic agents including KA when compared to the SD rats (30, 31), and this major sensitivity of WH might result in the higher mortality observed in this strain.

The latent phase to disease onset represents a crucial period of time to investigate mechanisms involved in epileptogenesis. Here, we have shown how this time window is significantly shorter in SD/H rats. During chronic epilepsy, SD/H rats displayed on average 15 times higher SRS frequency than WH/CR animals. These remarkable findings might suggest that the KASE WH/CR rats are more resistant to epilepsy or they are characterized by a slower disease progression.

The high divergence in SRS frequency of the two strains offers the possibility to choose the most appropriate strain to answer specific research questions. The WH/CR strain following modified protocol is characterized by a longer latent phase and low prevalence of SRS, meaning that it is useful to evaluate changes during epileptogenesis. For instance, such strain can be relevant in the identification and evaluation of risk (e.g., prenatal or early life stress) (32, 33) and precipitating factors (e.g., sleep deprivation) (34), which could aggravate epileptogenesis and seizure susceptibility. This model could be of great relevance in the evaluation of anti-epileptogenic treatments to halt or prevent epileptogenesis, especially to investigate the effect of anti-inflammatory treatments on the development of epilepsy. Given the strong microgliosis occurring in this model, it could be preferred in the evaluation of anti-inflammatory treatments. In addition, it is relevant to underline how WH/CR rats display HS similar to the majority of patient with TLE (type 1 HS). However, it is important to note that animal models with a more equal proportion of epileptic and non-epileptic animals would be needed to determine the specificity and sensitivity of biomarkers to predict acquired epilepsy (35, 36).

On the other hand, the SD/H strain is characterized by a short latent phase and high prevalence of SRS, which makes this strain appropriate for the evaluation of novel antiepileptic treatments or disease modification of existing epilepsy. The high SRS frequency would allow detecting more subtle effects of the treatment on the number and severity of SRS. Another feature of this strain is that all the animals subjected to KASE develop SRS as also confirmed by several independent *in-house* and previously published studies (22, 25). An important downside when performing intervention studies is the high variability in SRS frequency in this model. We observed that a proportion of animals was having very few seizures. This can be circumvented by pre-selection of a homogenous group of animals before entering the treatment study.

It is relevant to underline that several studies have demonstrated intra- and inter-strain variations in both focal and systemically induced TLE models regarding the SE and development of SRS (37–43). WH as well as SD rats are outbred strains with a high genetic heterogeneity and high intra-strain variation (37, 44). Moreover, epigenetic and environmental factors, including vendor-related effects, can also be important contributors in the intra-strain differences seen (37, 45).

The two rat strains present different pattern of neuronal loss. Although it may be expected that the rat strain with higher seizure burden would be characterized by more severe neuronal loss, it is important to underline that neuronal loss and seizure frequency are not univocally linked to each other as supported by previous studies (17, 46–49). Indeed, we observed that the KASE WH/CR rats had neuronal loss across all the investigated regions, while the KASE SD/H rats showed focal neuronal loss mainly in the PC. The two different patterns suggest neuronal loss could be the result of different processes. In particular, possible mechanisms involved in the extensive neuronal loss in CA1 of WH/CR animals could be a consequence of hypoxia and ischemia, which are known to occur during SE (50), and their effects could be more pronounced in this strain. Another possible explanation could be that WH/CR rats are more susceptible to the neurotoxic effect of KA and/or seizure-induced excitotoxicity, leading to increased cell loss as well as higher mortality.

The strong inflammatory response seen with both OX-42 and TSPO 1 week post-SE might be mainly driven by response to the primary injury, which was similar for both strains when using the modified induction protocol. This is supported by studies showing that high microglial activation is typically seen during the early phases of disease ontogenesis in different models of CNS injury (51–53). During the chronic period, the % area of microglial activation was overall reduced, however, still strongly increased in PC of both strains, and hippocampal sub-regions of WH/CR. In addition, chronic neurodegeneration was strikingly different between the two rat strains. The extensive neurodegeneration in WH/CR animals suggests the presence of abundant cell death, which might be the result or cause of the chronic microglial response seen in KASE WH/CR, explaining the higher TSPO focal binding seen in WH/CR compared to SD/H. Indeed, the high levels of cellular debris derived from the neuronal loss may trigger chronic microglial activation promoting neurodegeneration and establishing a vicious cycle that persists during chronic epilepsy. In accordance, a previous study (54) reported concurrent neurodegeneration and microgliosis in WH rats during chronic epilepsy in the KASE model of TLE. On the contrary, for SD/H rats, neuronal loss may be only related to SE insult, as previously shown (17), and the occurrence of SRS may result in a limited involvement of microglial activation and neurodegeneration in hippocampus as previously reported (48, 55). Furthermore, in agreement with our finding, neurodegeneration in the para-hippocampal regions has been reported until 3 months’ post-SE in the KASE model of TLE in SD rats (56).

In conclusion, we described two rat strains subjected to KASE, one with a low (WH/CR) and one with a high (SD/H) prevalence of SRS during the chronic period. The differences in disease progression and seizure outcome between strains may help to select the appropriate strain to study different research questions, including mechanisms of epileptogenesis, the search for prognostic factors, and the effect of treatments. In particular, these strain-related seizure phenotype and neuropathology indicate WH/CR as a more appropriate strain to investigate epileptogenesis, while SD/H to develop antiepileptic treatments. This work also confirms that in the same epilepsy model, the disease outcome can develop with different timing, features, and severity in different strains. This provides valuable information to reduce time, variables, and number of animals needed to achieve the specific research question in future studies. In addition, studying these diverging strains may lead to new insights regarding mechanisms underlying epilepsy.

## Ethics Statement

This study was carried out in accordance with the recommendations of the European Directive (2010/63/EU) on the protection of animals used for experimental and other scientific purposes. The protocol was approved (ECD 2014-39) by the ethical committee of the University of Antwerp (Belgium).

## Author Contributions

HA and SD conceived and designed the study. DB, HA, AE, RH, SV, and IA were involved in execution of the experimental design, data acquisition, and data interpretation for the study. DB, HA, IA, and SD were involved in drafting and editing the manuscript and figures. All authors approved the final manuscript and they are accountable for the content of the work.

## Conflict of Interest Statement

None of the authors has any conflict of interest to disclose. The authors declare that the research was conducted in the absence of any commercial or financial relationships that could be construed as a potential conflict of interest.

## Supplementary Material

Figure S1 Representative delineation of the investigated regions.

Figure S2 TSPO-specific binding 1 week post-SE in WH/CR and SD/H rats. For all brain regions and for both strains studied, a significant difference could be demonstrated between control and KASE animals 1 week post-SE. Kruskal–Wallis test with *post hoc* Dunn’s test. **p* < 0.05, ***p* < 0.01, ****p* < 0.001. KASE, kainic acid-induced *status epilepticus*; TSPO, translocator protein; status epilepticus, SE.

Figure S3 TSPO-specific binding 12 weeks post-SE in WH/CR and SD/H rats. In the KASE WH/CR animals, increased TSPO-specific binding 12 weeks post-SE could only be demonstrated in the CA1 when compared to control WH/CR rats. On the contrary, KASE SD/H rats showed a significant increase in all regions investigated compared to control SD/H animals. Kruskal–Wallis test with *post hoc* Dunn’s test. **p* < 0.05, ***p* < 0.01, ****p* < 0.001. KASE, kainic acid-induced *status epilepticus*; TSPO, translocator protein; status epilepticus, SE.

Figure S4 The number of fluorojade C (FjC)-positive cells inversely correlated with the number of neurons 1 week post-status epilepticus in WH/CR and SD/H rats. A statistically significant correlation between the number of FjC-positive cells and neurons was determined in all investigated regions. Spearman’s rank test.

Figure S5 The number of fluorojade C (FjC)-positive cells correlated with the % area of OX-42 1 week post-status epilepticus in WH/CR and SD/H rats. A statistically significant correlation between the % area of OX-42 and the number of FjC-positive cells was determined in all investigated regions. Spearman’s rank test.

## References

[1] Loscher W. Critical review of current animal models of seizures and epilepsy used in the discovery and development of new antiepileptic drugs. Seizure (2011) 20(5):359–68. 10.1016/j.seizure.2011.01.003 21292505

[2] Loscher W Klitgaard H Twyman RE Schmidt D. New avenues for anti-epileptic drug discovery and development. Nat Rev Drug Discov (2013) 12(10):757–76. 10.1038/nrd4126 24052047

[3] Levesque M Avoli M Bernard C. Animal models of temporal lobe epilepsy following systemic chemoconvulsant administration. J Neurosci Methods (2016) 260:45–52. 10.1016/j.jneumeth.2015.03.009 25769270 PMC4880459

[4] Morimoto K Fahnestock M Racine RJ. Kindling and status epilepticus models of epilepsy: rewiring the brain. Prog Neurobiol (2004) 73(1):1–60. 10.1016/j.pneurobio.2004.03.009 15193778

[5] Grone BP Baraban SC. Animal models in epilepsy research: legacies and new directions. Nat Neurosci (2015) 18(3):339–43. 10.1038/nn.3934 25710835

[6] Loscher W Brandt C High seizure frequency prior to antiepileptic treatment is a predictor of pharmacoresistant epilepsy in a rat model of temporal lobe epilepsy. Epilepsia (2010) 51(1):89–97. 10.1111/j.1528-1167.2009.02183.x 19563347

[7] Tellez-Zenteno JF Hernandez-Ronquillo L. A review of the epidemiology of temporal lobe epilepsy. Epilepsy Res Treat (2012) 2012:630853. 10.1155/2012/630853 22957234 PMC3420432

[8] Hellier JL Patrylo PR Buckmaster PS Dudek FE. Recurrent spontaneous motor seizures after repeated low-dose systemic treatment with kainate: assessment of a rat model of temporal lobe epilepsy. Epilepsy Res (1998) 31(1):73–84. 10.1016/S0920-1211(98)00017-5 9696302

[9] Doelken MT Stefan H Pauli E Stadlbauer A Struffert T Engelhorn T (1)H-MRS profile in MRI positive- versus MRI negative patients with temporal lobe epilepsy. Seizure (2008) 17(6):490–7. 10.1016/j.seizure.2008.01.008 18337128

[10] Nairismagi J Pitkanen A Kettunen MI Kauppinen RA Kubova H. Status epilepticus in 12-day-old rats leads to temporal lobe neurodegeneration and volume reduction: a histologic and MRI study. Epilepsia (2006) 47(3):479–88. 10.1111/j.1528-1167.2006.00455.x 16529609

[11] Vivash L Gregoire MC Bouilleret V Berard A Wimberley C Binns D In vivo measurement of hippocampal GABAA/cBZR density with [18F]-flumazenil PET for the study of disease progression in an animal model of temporal lobe epilepsy. PLoS One (2014) 9(1):e86722. 10.1371/journal.pone.0086722 24466212 PMC3897736

[12] Thom M. Review: hippocampal sclerosis in epilepsy: a neuropathology review. Neuropathol Appl Neurobiol (2014) 40(5):520–43. 10.1111/nan.12150 24762203 PMC4265206

[13] Dedeurwaerdere S Fang K Chow M Shen YT Noordman I van Raay L Manganese-enhanced MRI reflects seizure outcome in a model for mesial temporal lobe epilepsy. Neuroimage (2013) 68:30–8. 10.1016/j.neuroimage.2012.11.054 23220429

[14] Bertoglio D Verhaeghe J Santermans E Amhaoul H Jonckers E Wyffels L Non-invasive PET imaging of brain inflammation at disease onset predicts spontaneous recurrent seizures and reflects comorbidities. Brain Behav Immun (2017) 61:69–79. 10.1016/j.bbi.2016.12.015 28017648

[15] Amhaoul H Hamaide J Bertoglio D Reichel SN Verhaeghe J Geerts E Brain inflammation in a chronic epilepsy model: evolving pattern of the translocator protein during epileptogenesis. Neurobiol Dis (2015) 82:526–39. 10.1016/j.nbd.2015.09.004 26388398

[16] Vivash L Tostevin A Liu DS Dalic L Dedeurwaerdere S Hicks RJ Changes in hippocampal GABAA/cBZR density during limbic epileptogenesis: relationship to cell loss and mossy fibre sprouting. Neurobiol Dis (2011) 41(2):227–36. 10.1016/j.nbd.2010.08.021 20816783

[17] Gorter JA Goncalves Pereira PM van Vliet EA Aronica E Lopes da Silva FH Lucassen PJ Neuronal cell death in a rat model for mesial temporal lobe epilepsy is induced by the initial status epilepticus and not by later repeated spontaneous seizures. Epilepsia (2003) 44(5):647–58. 10.1046/j.1528-1157.2003.53902.x 12752463

[18] Akiyama H Tooyama I Kondo H Ikeda K Kimura H McGeer EG Early response of brain resident microglia to kainic acid-induced hippocampal lesions. Brain Res (1994) 635(1–2):257–68. 10.1016/0006-8993(94)91447-8 8173962

[19] van Vliet EA da Costa Araujo S Redeker S van Schaik R Aronica E Gorter JA. Blood-brain barrier leakage may lead to progression of temporal lobe epilepsy. Brain (2007) 130(Pt 2):521–34. 10.1093/brain/awl318 17124188

[20] van Vliet EA Holtman L Aronica E Schmitz LJ Wadman WJ Gorter JA. Atorvastatin treatment during epileptogenesis in a rat model for temporal lobe epilepsy. Epilepsia (2011) 52(7):1319–30. 10.1111/j.1528-1167.2011.03073.x 21729039

[21] Van Nieuwenhuyse B Raedt R Sprengers M Dauwe I Gadeyne S Carrette E The systemic kainic acid rat model of temporal lobe epilepsy: long-term EEG monitoring. Brain Res (2015) 1627:1–11. 10.1016/j.brainres.2015.08.016 26381287

[22] Van Nieuwenhuyse B Raedt R Delbeke J Wadman WJ Boon P Vonck K. In search of optimal DBS paradigms to treat epilepsy: bilateral versus unilateral hippocampal stimulation in a rat model for temporal lobe epilepsy. Brain Stimul (2015) 8(2):192–9. 10.1016/j.brs.2014.11.016 25554585

[23] Powell KL Ng C O’Brien TJ Xu SH Williams DA Foote SJ Decreases in HCN mRNA expression in the hippocampus after kindling and status epilepticus in adult rats. Epilepsia (2008) 49(10):1686–95. 10.1111/j.1528-1167.2008.01593.x 18397293

[24] Dedeurwaerdere S Callaghan PD Pham T Rahardjo GL Amhaoul H Berghofer P PET imaging of brain inflammation during early epileptogenesis in a rat model of temporal lobe epilepsy. EJNMMI Res (2012) 2(1):60. 10.1186/2191-219X-2-60 23136853 PMC3570346

[25] Amhaoul H Ali I Mola M Van Eetveldt A Szewczyk K Missault S P2X7 receptor antagonism reduces the severity of spontaneous seizures in a chronic model of temporal lobe epilepsy. Neuropharmacology (2016) 105:175–85. 10.1016/j.neuropharm.2016.01.018 26775823

[26] Racine RJ Modification of seizure activity by electrical stimulation. II. Motor seizure. Electroencephalogr Clin Neurophysiol (1972) 32(3):281–94. 10.1016/0013-4694(72)90176-9 4110397

[27] Paxinos C Watson C The Rat Brain in Stereotaxic Coordinates. Elsevier Inc. (2007).

[28] Schmued LC Stowers CC Scallet AC Xu L. Fluoro-Jade C results in ultra high resolution and contrast labeling of degenerating neurons. Brain Res (2005) 1035(1):24–31. 10.1016/j.brainres.2004.11.054 15713273

[29] Tedesco V Ravagnani C Bertoglio D Chiamulera C. Acute ketamine-induced neuroplasticity: ribosomal protein S6 phosphorylation expression in drug addiction-related rat brain areas. Neuroreport (2013) 24(7):388–93. 10.1097/WNR.0b013e32836131ad 23568219

[30] Golden GT Smith GG Ferraro TN Reyes PF Kulp JK Fariello RG. Strain differences in convulsive response to the excitotoxin kainic acid. Neuroreport (1991) 2(3):141–4. 10.1097/00001756-199103000-00008 1768857

[31] Golden GT Smith GG Ferraro TN Reyes PF. Rat strain and age differences in kainic acid induced seizures. Epilepsy Res (1995) 20(2):151–9. 10.1016/0920-1211(94)00079-C 7750511

[32] Ali I O’Brien P Kumar G Zheng T Jones NC Pinault D Enduring effects of early life stress on firing patterns of hippocampal and thalamocortical neurons in rats: implications for limbic epilepsy. PLoS One (2013) 8(6):e66962. 10.1371/journal.pone.0066962 23825595 PMC3688984

[33] Yin P Liu J Li Z Wang YY Qiao NN Huang SY Prenatal immune challenge in rats increases susceptibility to seizure-induced brain injury in adulthood. Brain Res (2013) 1519:78–86. 10.1016/j.brainres.2013.04.047 23648360

[34] Matos G Scorza FA Mazzotti DR Guindalini C Cavalheiro EA Tufik S The effects of sleep deprivation on microRNA expression in rats submitted to pilocarpine-induced status epilepticus. Prog Neuropsychopharmacol Biol Psychiatry (2014) 51:159–65. 10.1016/j.pnpbp.2014.02.001 24530830

[35] Choy M Dube CM Patterson K Barnes SR Maras P Blood AB A novel, noninvasive, predictive epilepsy biomarker with clinical potential. J Neurosci (2014) 34(26):8672–84. 10.1523/JNEUROSCI.4806-13.2014 24966369 PMC4069350

[36] Pascente R Frigerio F Rizzi M Porcu L Boido M Davids J Cognitive deficits and brain myo-Inositol are early biomarkers of epileptogenesis in a rat model of epilepsy. Neurobiol Dis (2016) 93:146–55. 10.1016/j.nbd.2016.05.001 27173096

[37] Portelli J Aourz N De Bundel D Meurs A Smolders I Michotte Y Intrastrain differences in seizure susceptibility, pharmacological response and basal neurochemistry of Wistar rats. Epilepsy Res (2009) 87(2–3):234–46. 10.1016/j.eplepsyres.2009.09.009 19833479

[38] Langer M Brandt C Loscher W. Marked strain and substrain differences in induction of status epilepticus and subsequent development of neurodegeneration, epilepsy, and behavioral alterations in rats. [corrected]. Epilepsy Res (2011) 96(3):207–24. 10.1016/j.eplepsyres.2011.06.005 21723093

[39] Brandt C Bankstahl M Tollner K Klee R Loscher W. The pilocarpine model of temporal lobe epilepsy: marked intrastrain differences in female Sprague-Dawley rats and the effect of estrous cycle. Epilepsy Behav (2016) 61:141–52. 10.1016/j.yebeh.2016.05.020 27344503

[40] Loscher W Ferland RJ Ferraro TN. The relevance of inter- and intrastrain differences in mice and rats and their implications for models of seizures and epilepsy. Epilepsy Behav (2017) 73:214–35. 10.1016/j.yebeh.2017.05.040 28651171 PMC5909069

[41] Hort J Brozek G Komarek V Langmeier M Mares P. Interstrain differences in cognitive functions in rats in relation to status epilepticus. Behav Brain Res (2000) 112(1–2):77–83. 10.1016/S0166-4328(00)00163-7 10862938

[42] Becker A Krug M Schroder H. Strain differences in pentylenetetrazol-kindling development and subsequent potentiation effects. Brain Res (1997) 763(1):87–92. 10.1016/S0006-8993(97)00409-5 9272832

[43] Schauwecker PE. Strain differences in seizure-induced cell death following pilocarpine-induced status epilepticus. Neurobiol Dis (2012) 45(1):297–304. 10.1016/j.nbd.2011.08.013 21878392 PMC3225715

[44] Yilmazer-Hanke DM. Morphological correlates of emotional and cognitive behaviour: insights from studies on inbred and outbred rodent strains and their crosses. Behav Pharmacol (2008) 19(5–6):403–34. 10.1097/FBP.0b013e32830dc0de 18690101

[45] Schridde U Strauss U Brauer AU van Luijtelaar G. Environmental manipulations early in development alter seizure activity, Ih and HCN1 protein expression later in life. Eur J Neurosci (2006) 23(12):3346–58. 10.1111/j.1460-9568.2006.04865.x 16820024

[46] Dingledine R Varvel NH Dudek FE. When and how do seizures kill neurons, and is cell death relevant to epileptogenesis? Adv Exp Med Biol (2014) 813:109–22. 10.1007/978-94-017-8914-1_9 25012371 PMC4624106

[47] Mathern GW Adelson PD Cahan LD Leite JP. Hippocampal neuron damage in human epilepsy: Meyer’s hypothesis revisited. Prog Brain Res (2002) 135:237–51. 10.1016/S0079-6123(02)35023-4 12143344

[48] Pitkanen A Nissinen J Nairismagi J Lukasiuk K Grohn OH Miettinen R Progression of neuronal damage after status epilepticus and during spontaneous seizures in a rat model of temporal lobe epilepsy. Prog Brain Res (2002) 135:67–83. 10.1016/S0079-6123(02)35008-8 12143371

[49] Arida RM Scorza FA de Amorim Carvalho R Cavalheiro EA. *Proechimys guyannensis*: an animal model of resistance to epilepsy. Epilepsia (2005) 46(Suppl 5):189–97. 10.1111/j.1528-1167.2005.01065.x 15987276

[50] Lucchi C Vinet J Meletti S Biagini G. Ischemic-hypoxic mechanisms leading to hippocampal dysfunction as a consequence of status epilepticus. Epilepsy Behav (2015) 49:47–54. 10.1016/j.yebeh.2015.04.003 25934585

[51] Jack C Ruffini F Bar-Or A Antel JP Microglia and multiple sclerosis. J Neurosci Res (2005) 81(3):363–73. 10.1002/jnr.20482 15948188

[52] Loane DJ Byrnes KR. Role of microglia in neurotrauma. Neurotherapeutics (2010) 7(4):366–77. 10.1016/j.nurt.2010.07.002 20880501 PMC2948548

[53] Weinstein JR Koerner IP Moller T. Microglia in ischemic brain injury. Future Neurol (2010) 5(2):227–46. 10.2217/fnl.10.1 20401171 PMC2853969

[54] Immonen RJ Kharatishvili I Sierra A Einula C Pitkanen A Grohn OH Manganese enhanced MRI detects mossy fiber sprouting rather than neurodegeneration, gliosis or seizure-activity in the epileptic rat hippocampus. Neuroimage (2008) 40(4):1718–30. 10.1016/j.neuroimage.2008.01.042 18328732

[55] Hopkins KJ Wang G Schmued LC Temporal progression of kainic acid induced neuronal and myelin degeneration in the rat forebrain. Brain Res (2000) 864(1):69–80. 10.1016/S0006-8993(00)02137-5 10793188

[56] Drexel M Preidt AP Sperk G. Sequel of spontaneous seizures after kainic acid-induced status epilepticus and associated neuropathological changes in the subiculum and entorhinal cortex. Neuropharmacology (2012) 63(5):806–17. 10.1016/j.neuropharm.2012.06.009 22722023 PMC3409872

