# Supplementary material for: Kainic Acid-Induced Post-Status Epilepticus Models of Temporal Lobe Epilepsy with Diverging Seizure Phenotype and Neuropathology
Source: Front Neurol. 2017 Nov 6;8:588. doi: 10.3389/fneur.2017.00588 (PMC5681498; doi:10.3389/fneur.2017.00588)
Supplement: Supplementary file 1 [file Table_1.DOCX]

Supplementary Material

**Kainic acid-induced post-status epilepticus models of temporal lobe epilepsy with diverging seizure phenotype and neuropathology**

**Daniele Bertoglio^1§^, Halima Amhaoul^1§^, Annemie Van Eetveldt^1^, Ruben Houbrechts^1^, Sebastiaan Van De Vijver^1^, Idrish Ali^1^, Stefanie Dedeurwaerdere^1,#,*^**

*^1^ Department of Translational Neurosciences, University of Antwerp, Belgium*

*^§^ Both authors contributed equally to this work.*

*** Correspondence:**

Dr. Stefanie Dedeurwaerdere

[Stefanie.dedeurwaerdere@hotmail.com](mailto:Stefanie.dedeurwaerdere@hotmail.com)

^#^ Current address: UCB Pharma, Rue du Foriest, 1420 Braine l-Alleud, Belgium

# Supplementary Figures and Tables

## Supplementary Tables
