# Supplementary figures and images for: Kainic Acid-Induced Post-Status Epilepticus Models of Temporal Lobe Epilepsy with Diverging Seizure Phenotype and Neuropathology
Source: Front Neurol. 2017 Nov 6;8:588. doi: 10.3389/fneur.2017.00588 (PMC5681498; doi:10.3389/fneur.2017.00588)

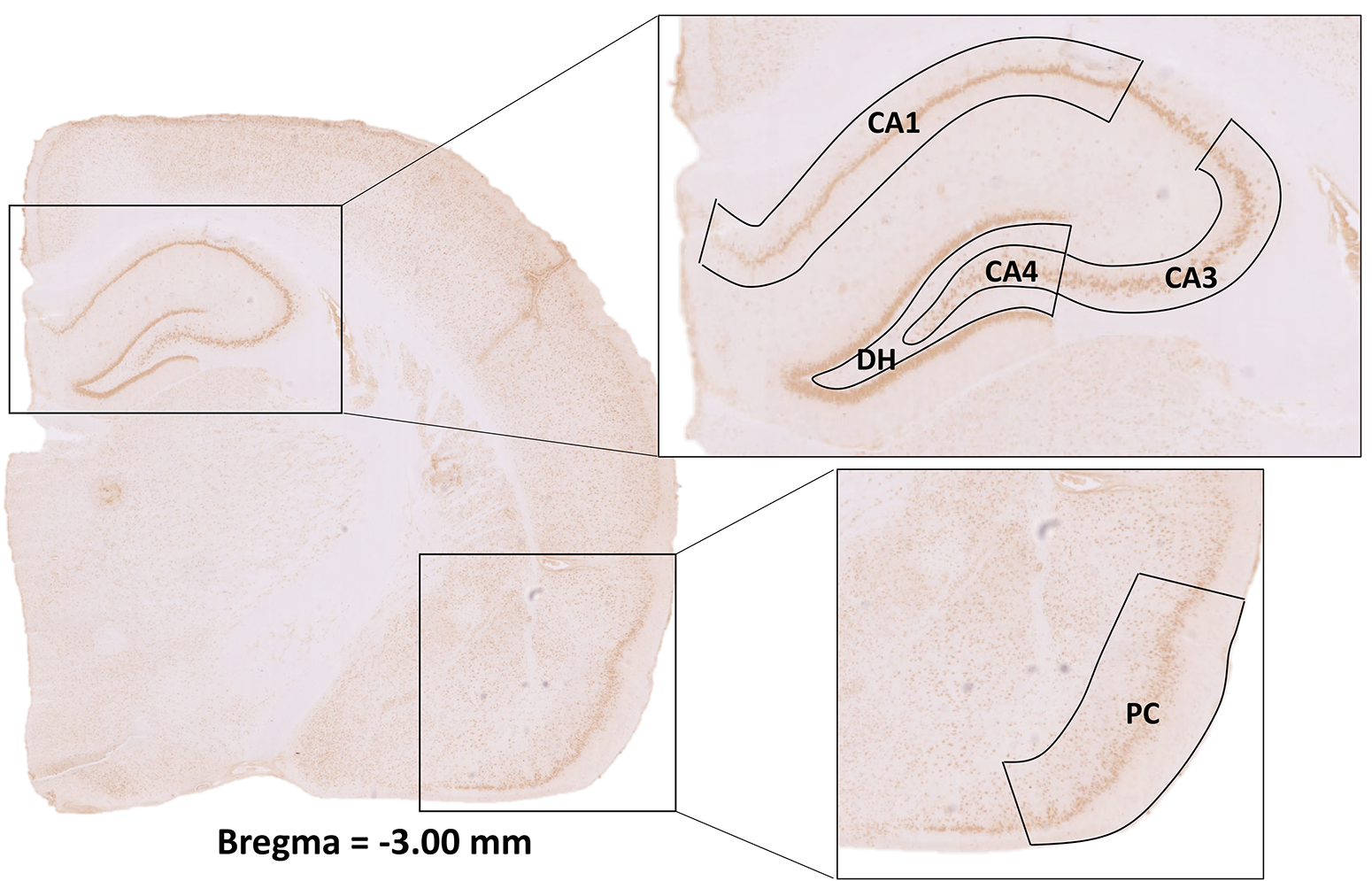

Supplement: Figure S1 — Representative delineation of the investigated regions. [file Image_1.TIF]

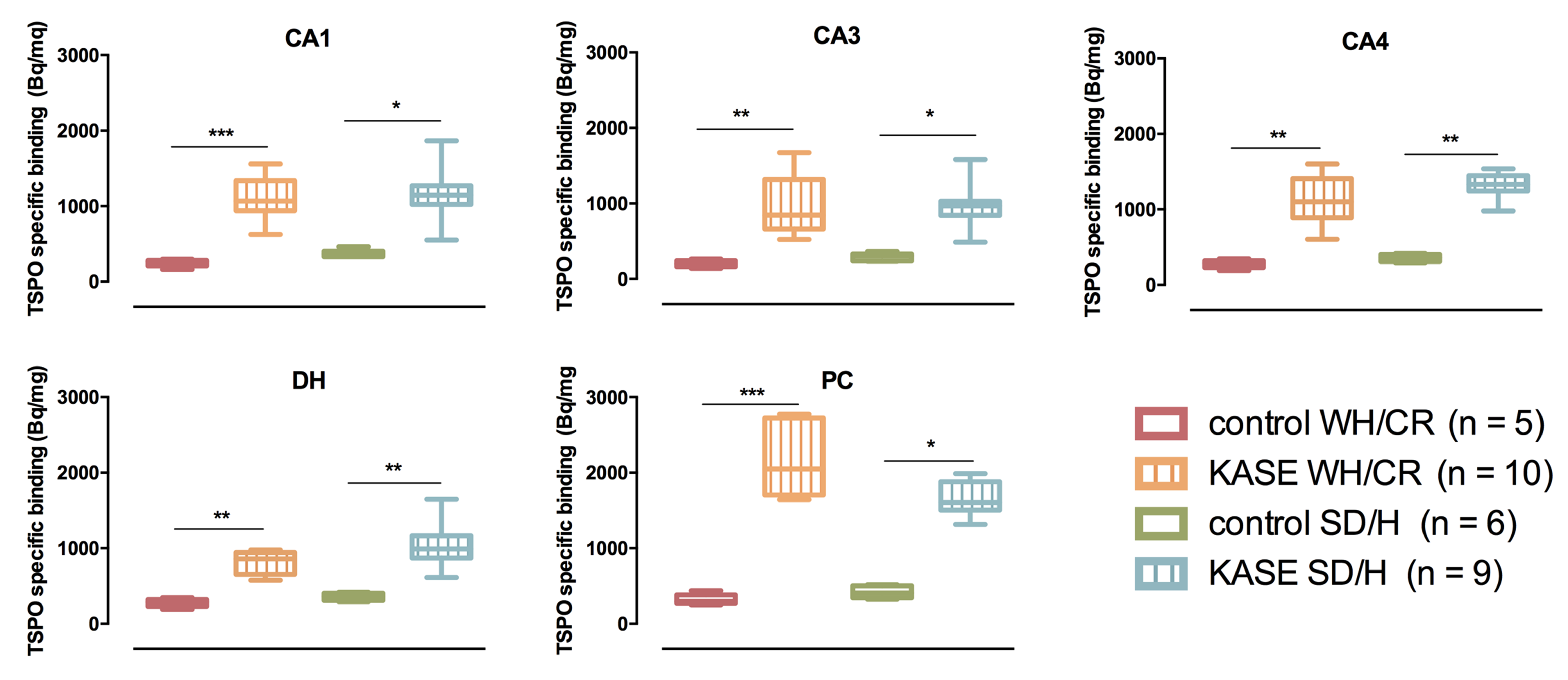

Supplement: Figure S2 — TSPO-specific binding 1 week post-SE in WH/CR and SD/H rats. For all brain regions and for both strains studied, a significant difference could be demonstrated between control and KASE animals 1 week post-SE. Kruskal–Wallis test with post hoc Dunn’s test. *p < 0.05, **p < 0.01, ***p < 0.001. KASE, kainic acid-induced status epilepticus; TSPO, translocator protein; status epilepticus, SE. [file Image_2.TIFF]

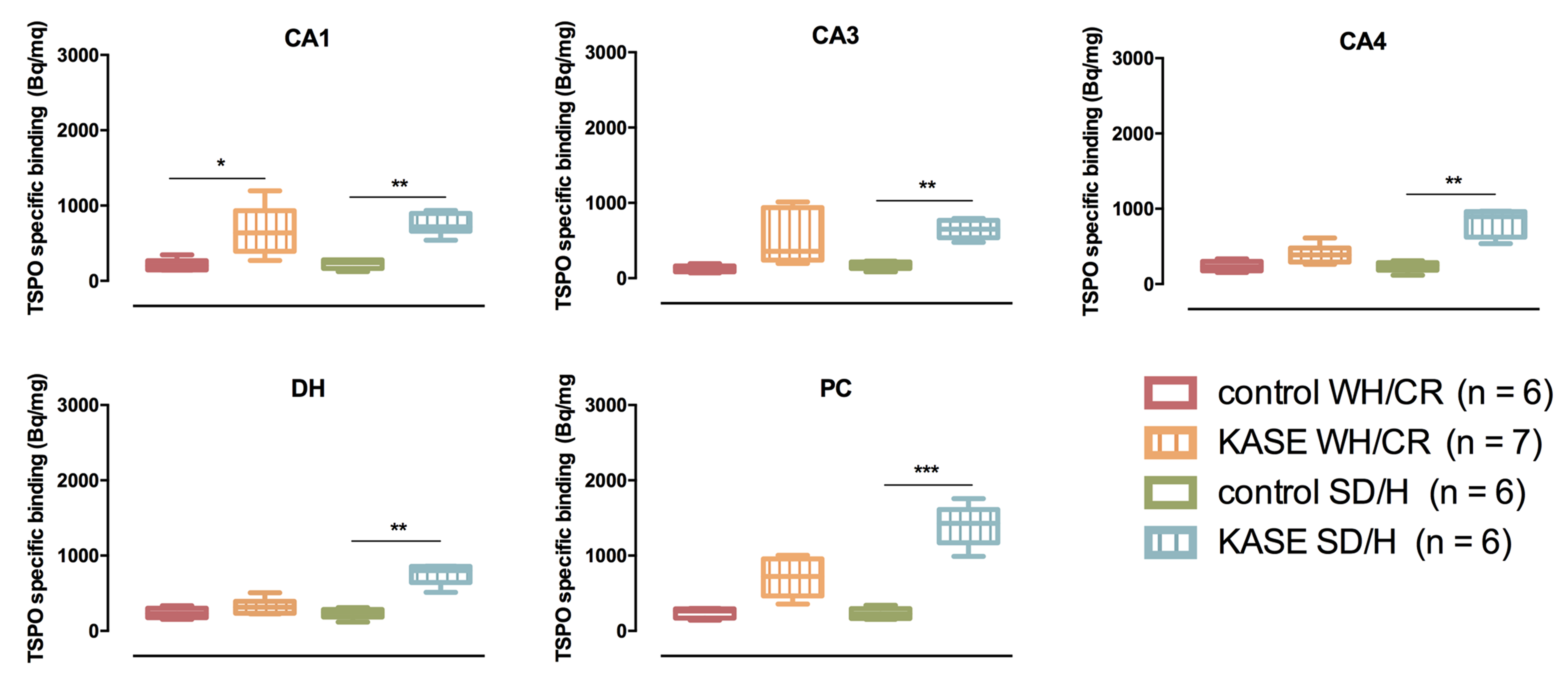

Supplement: Figure S3 — TSPO-specific binding 12 weeks post-SE in WH/CR and SD/H rats. In the KASE WH/CR animals, increased TSPO-specific binding 12 weeks post-SE could only be demonstrated in the CA1 when compared to control WH/CR rats. On the contrary, KASE SD/H rats showed a significant increase in all regions investigated compared to control SD/H animals. Kruskal–Wallis test with post hoc Dunn’s test. *p < 0.05, **p < 0.01, ***p < 0.001. KASE, kainic acid-induced status epilepticus; TSPO, translocator protein; status epilepticus, SE. [file Image_3.TIFF]

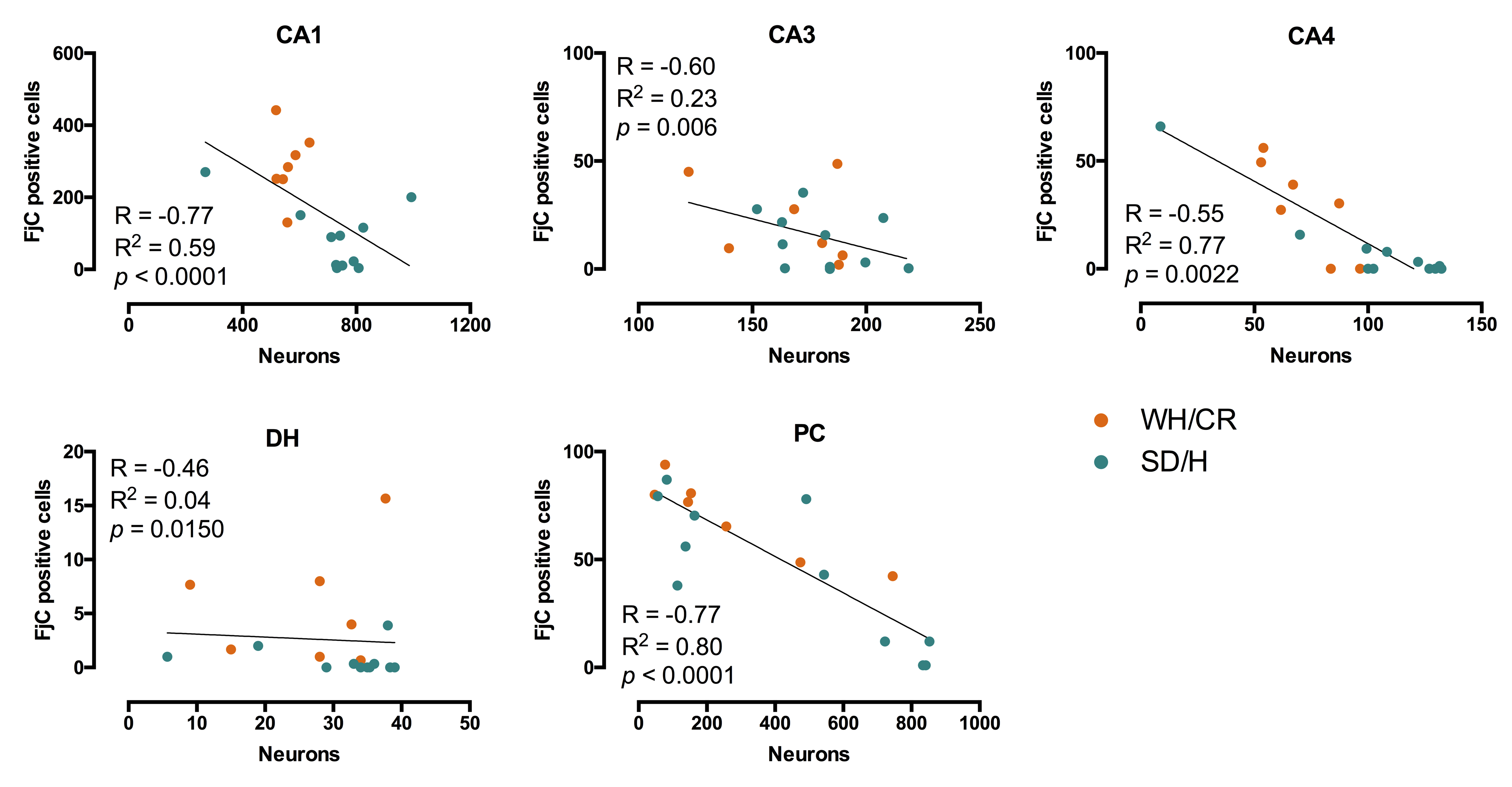

Supplement: Figure S4 — The number of fluorojade C (FjC)-positive cells inversely correlated with the number of neurons 1 week post-status epilepticus in WH/CR and SD/H rats. A statistically significant correlation between the number of FjC-positive cells and neurons was determined in all investigated regions. Spearman’s rank test. [file Image_4.TIFF]

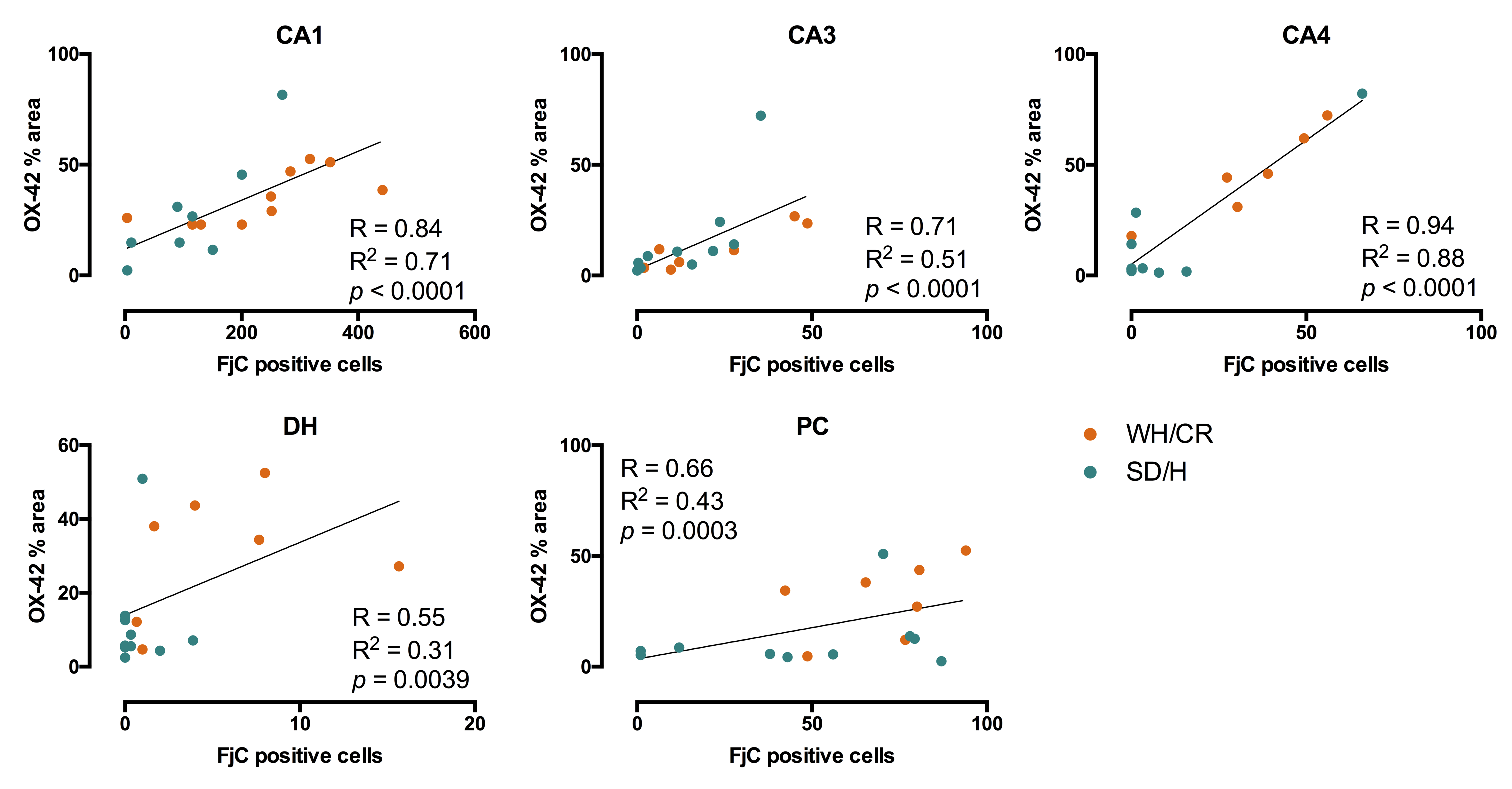

Supplement: Figure S5 — The number of fluorojade C (FjC)-positive cells correlated with the % area of OX-42 1 week post-status epilepticus in WH/CR and SD/H rats. A statistically significant correlation between the % area of OX-42 and the number of FjC-positive cells was determined in all investigated regions. Spearman’s rank test. [file Image_5.TIFF]
